# Knowledge of obstetric fistulas and associated factors among women of reproductive age in Ethiopia: Systematic review and meta‐analysis

**DOI:** 10.1002/ijgo.70566

**Published:** 2025-09-30

**Authors:** Aster Shiferaw, Getachew Tilaye Mihiret, Mastewal Yechale Mihret

**Affiliations:** ^1^ Department of Midwifery, College of Medicine and Health Science Debre Markos University Debre Markos Amhara Region Ethiopia

**Keywords:** associated factors, Ethiopia, knowledge, obstetric fistula, reproductive age women

## Abstract

**Objectives:**

Obstetric fistula is a complication occurring in childbearing women. It is a major problem in developing countries and results in poor childhood development and limited use of obstetric care. The aim of this study was to show the pooled prevalence of knowledge of obstetric fistulas among reproductive age women.

**Method:**

Several databases and websites were searched to find articles. Studies conducted on the knowledge of obstetric fistula and associated factors in women of reproductive age in Ethiopia up to February 20, 2023, were included. Data collection and analysis: The Joanna Briggs Institute Meta‐Analysis of Statistics Assessment and Review Instrument for cross‐sectional study was used for quality assessment, and the Preferred Reporting Items for Systematic reviews and Meta‐Analysis guidelines were used for review. Seven studies were included in total. Pooled prevalence was calculated using a random effect model, and subgroup analysis was carried out. Egger's and Begg's tests were used to assess for publication bias. Finally, tests were conducted to determine the impact of related factors on obstetric fistula knowledge.

**Results:**

The pooled prevalence of knowledge of obstetric fistula among reproductive age women was 43.9%. Attending formal education (AOR = 3.74, 95% confidence interval [CI] = 1.43, 6.05), urban residence (AOR = 4.65, 95% CI = 2.79, 6.52), having antenatal care (ANC) history (AOR = 5.69, 95% CI = 2.03, 9.3), having family planning (FP) history (AOR = 2.5, 95% CI = 1.11, 3.9), home distance from health institution that took ≤30 min by foot (AOR = 3.85, 95% CI = 2.47, 5.23), and ever having been pregnant (AOR = 2.68, 95% CI = 1.25, 4.11).

**Conclusion:**

In this study, most women of reproductive age did not know anything about obstetric fistulas. Knowledge about obstetric fistulas was strongly associated with living in an urban area, walking ≤30 min to and from a medical facility, having a history of ANC or FP, and having ever been pregnant. Therefore, it is important to advocate for women's education, access to maternity and child health services, communication with medical professionals, and proximity to health facilities.

**Abbreviations**
ANC: antenatal care
AOR: Adjusted odd ratio
CI: Confidence interval
EMOH: Ethiopian minister of health
FP: family planning
JBI‐MAStARI: Joanna Briggs Institute Meta‐Analysis of Statistics Assessment and Review Instrument
MCH: maternity and child health
PRISMA: Preferred reporting item for systematic review and meta‐ analysis
RVF: recto‐vaginal fistula
SNNP: south nation nationalities and peoples
SSA: sub‐Saharan Africa
UVF: urethra‐vaginal fistula
VVF: Volvo‐vaginal fistula

## 1 BACKGROUND

An obstetric fistula is an abnormal opening or connection between a woman's genital tract and urinary tract or genital tract and gastrointestinal tract that leads to a woman having continuous leakage of stool or urine. There are different types of obstetric fistulas, including vesico‐vaginal fistulas (VVF), where an opening occurs between the bladder and vagina; urethra‐vaginal fistulas (UVF), where an opening occurs between the urethra and vagina; recto‐vaginal fistulas (RVF), where an opening occurs between the rectum and vagina; uretero‐vaginal fistulas, which occur between the ureters and vagina; and vesico‐uterine fistulas, which occur between the bladder and uterus. A fistula generally develops due to the compression of soft tissue between hard bones of the maternal pelvis and fetal head during uterine contraction, which results in ischemia of tissue due to restriction oxygen supply and tearing of soft tissue during precipitated delivery or obstetric maneuvers/use of instruments. The most common cause of obstetric fistulas is obstructed or prolonged labor. Other causes include cancer, radiation treatment for cancer, injury from gynecologic or obstetric surgery, coital trauma, sexual violence, infections like lymphogranuloma venereum, and female genital mutilation.^1^, ^2^, ^3^, ^4^

Obstetric fistulas are a complication of childbearing. In developing countries, most fistulas result from poor childhood development and limited availability and consumption of obstetric care. Worldwide, approximately 2–3 million women and approximately 2 million young women in sub‐Saharan Africa and South Asia are affected by obstetric fistulas. Globally, between 50 000 and 100 000 new instances of fistulas have been reported annually, with between 30 000 and 130 000 new cases occurring in sub‐Saharan Africa. While there has been a decrease in the percentage of women with obstetric fistulas, from 2010 to 2013, approximately 2000 women underwent surgical intervention for obstetric fistulas and over 110 000 women, on average, had vaginal fistulas in Ethiopia annually.^4^, ^5^, ^6^, ^7^, ^8^

Obstetric fistulas cause women to have severe and uncontrollable multi‐dimensional health problems, with psycho‐social consequences, including being abandoned, being divorced, becoming childless, being stigmatized, and being shunned by their community.^9^ Women are criticized and accused of being exposed to fistulas as a punishment for their sins or for contracting venereal disease. Women with fistulas can be restricted from participating in religious activities and social events. Loss of hope and dignity, lack of support and power to seek care, fear and distress about their future lives, and feelings of dependency are major mental health problems resulting from fistulas. Women find themselves facing an absence of support and family care, physical and economic inability to access health care and a lack of information and knowledge about fistula care and treatment.^9^, ^10^, ^11^, ^12^, ^13^, ^14^, ^15^ Even though studies have been conducted on knowledge and awareness of obstetric fistulas,^16^, ^17^, ^18^ published data on the knowledge of obstetric fistulas among women of reproductive age in Ethiopia is scarce. Therefore, this systematic review and meta‐analysis provides information about the knowledge of women on obstetric fistulas and associated factors in Ethiopia. This study might help the responsible bodies to take appropriate measures to improve the knowledge of women about obstetric fistulas and address the contributing factors. Women of reproductive age having knowledge about obstetric fistulas helps in establishing prevention measures before the problem occurs. Women become alert for contributing factors and can identify problems. Further, they access health care early, preventing exposure to further morbidity and mortality.

## 2 METHODS

### 2.1 Study design and research strategies

This systematic review and meta‐analysis was conducted using published articles on the knowledge of obstetric fistulas and associated factors among women of reproductive age in Ethiopia. The presence of systematic reviews and meta‐analyses on this topic was checked to prevent duplication. Then articles were searched online through Google Scholar, PubMed/MEDLINE, Cochrane Library, EMBASE, HINARI, African journals and institutional repositories, and the reference list of searched articles was exported to endnote. Thus, research articles were reviewed systematically following Preferred Reporting Items for Systematic review and Meta‐Analysis (PRISMA) guidelines^19^ and analyzed to show the magnitude of women of reproductive ages' knowledge of obstetric fistulas and associated factors. “Knowledge,” “awareness,” “obstetric fistula,” “associated factors,” “reproductive age women,” and “Ethiopia” were the key terms used to search articles. The search was conducted from January 10 to February 20, 2023.

### 2.2 Inclusion criteria

All published studies conducted on the awareness/knowledge of obstetric fistulas and associated factors among women of reproductive age in Ethiopia until February 20, 2023, were included.

### 2.3 Exclusion criteria

Articles in languages other than English and that did not measure the outcome of the review were excluded.

### 2.4 Data extraction

The articles were searched, collected, and exported to endnote version 7.1. Repeated articles were removed. The data were extracted using the 2014 Joanna Briggs Institute Reviewers' Manual data extraction form, which includes the title, author, year of study, year of publication, study design, sample size, study participants, study area, response rate, sampling method, the magnitude of knowledge of obstetric fistulas, and factors associated with knowledge of obstetric fistula.

### 2.5 Outcome measurement

This systematic review and meta‐analysis had two outcomes: the first one was pooled prevalence of women's knowledge of obstetric fistula. Women's knowledge of obstetric fistulas was assessed using items focusing on information about obstetric fistula cause, prevention, presentation, and treatment options. The second outcome was the factors associated with the knowledge of obstetric fistula. Factors repeatedly reported from previous studies were level of education, having antenatal care (ANC) history, having family planning (FP) history, ever having been pregnant, home distance from health institution, residence, prior information of fistulas, participation in conferences, place of delivery, and having heard about obstetric complications.^16^, ^17^, ^18^, ^20^, ^21^

### 2.6 Quality assessment and data collection

The searched articles were critically appraised based on the Joanna Briggs Institute Meta‐Analysis of Statistics Assessment and Review Instrument (JBI‐MAStARI) checklist for cross‐sectional studies.^22^ The criteria used to evaluate the quality of each article were: “Were the criteria for inclusion in the sample clearly defined?”, “Were the study subjects and the setting described in detail?”, “Was the exposure measured in a valid and reliable way?”, “Were objective, standard criteria used for measurement of the condition?”, “Were confounding factors identified?”, “Were strategies to deal with confounding factors stated?”, “Were the outcomes measured in a valid and reliable way?”, and “Was appropriate statistical analysis used?” Each article was reviewed independently by three reviewers. They then rechecked each other's articles and reassessed again to solve the inconsistencies and the differences between the reviews. The reviewers engaged in dialogue until all the reviewers had reached full agreement. All articles were evaluated for their quality before being included in the final review by all authors. Articles with a critical appraisal score 50% and above based on listed criteria were approved for the final systematic review and meta‐analysis.^23^

### 2.7 Data analysis

A Microsoft Excel spreadsheet was used to enter the extracted data and was exported to STATA version 17 for analysis. The presence of heterogeneity was assessed using Cochran's Q statistics and presented with inverse variance (*I* ^2^). The result is interpreted as follows: 25%, 50%, and 75% as low, medium, and high heterogeneity, respectively, at *P*‐values <0.05.^24^ Heterogeneity might occur due to the methodology, randomization, differences in datasets across the studies, and publication bias in the studies. Begg‐Mazumdar rank correlation and Egger's regression tests were used to assess the significant correlation between effect estimates, and their variance at *P*‐values <0.05 were used to check the presence of publication bias.^25^ A random effects meta‐analysis model was used to show the pooled prevalence of knowledge of obstetric fistulas and presented in a forest plot with the corresponding 95% CI. The pooled odds ratio was used to determine the presence of a significant association between the knowledge of obstetric fistulas and associated factors.

## 3 RESULTS

### 3.1 Results of search

**FIGURE 1:**
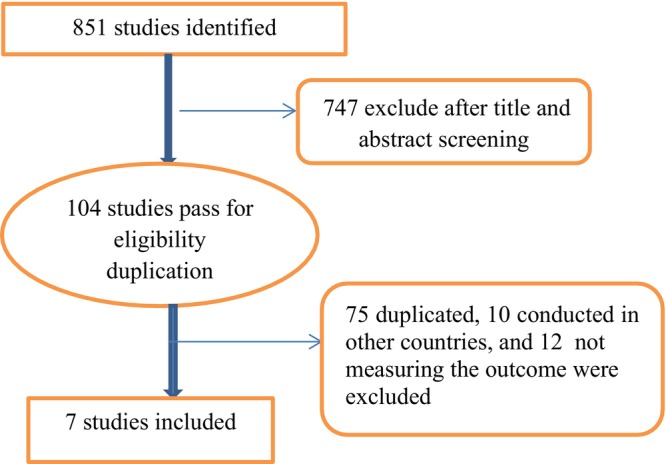
Diagrammatic presentation of searched articles.

A total of 851 research articles were identified through different search databases. Among those, 747 were excluded after screening of titles and abstracts. The remaining 104 were transferred to check duplication and eligibility; then 10 studies that were conducted in other countries and 12 studies that did not measure the outcome of this systematic review and meta‐analysis were excluded. Again, 75 duplicated articles were removed using endnotes and, finally, seven studies were included in this study (Figure 1).

### 3.2 Characteristics of the study

Seven studies were included, and a total of 3413 study participants were involved in this systematic review and meta‐analysis. The sample sizes of studies evaluated ranged from 400 to 773. Three studies were conducted in the Amhara region: one in Oromia, one in Tigray, one in the Southern Nations, Nationalities, and Peoples' Region (SNNPR), and one in southwest Ethiopia. All studies were cross‐sectional and conducted from 2018 to 2022. Five studies were community based, and the other two were conducted in health institutions (Table 1).

**TABLE 1:** Characteristics of the included studies.

Number	Author	Region	Study year	Study design	Sampling technique	Sample size	Outcome size	Prevalence	SeP	Quality score (%)
1	Semira Defar	Oromia	2018	Community‐based cross‐sectional	Systematic random	400	200	50	2.5	100
2	Samrawit, Emily	Amhara	2022	Community based cross‐sectional	Systematic random	400	196	49	2.5	100
3	Wondu Feyisa Balcha et al.	Amhara	2019	Institution‐based cross‐sectional	Systematic random	413	163	39.5	2.40	75
4	Asefa Z et al.	SNNP	2020	Community‐based cross‐sectional	Systematic random	422	172	40.8	2.39	75
5	Rundasa et al.	South west	2021	Institution‐based cross‐sectional	Simple random	400	200	50	2.5	75
6	Tsega Dejen et al.	Amhara	2021	Community‐based cross‐sectional	Systematic random	773	281	36.6	1.73	75
7	Berhane Teklay et al.	Tigray	2020	Community‐based cross‐sectional	Multi‐stage	605	255	42.15	2.01	100

Abbreviation: SeP, standard error of prevalence.

### 3.3 Pooled prevalence of knowledge of obstetric fistula

**FIGURE 2:**
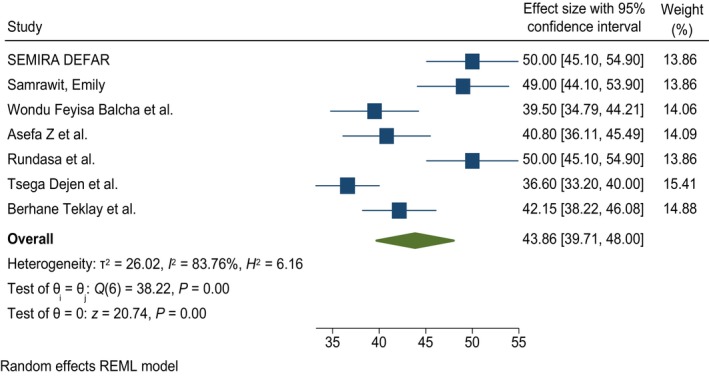
Pooled magnitude of knowledge of obstetric fistula in Ethiopia. REML, random effect model.

Cochran's *Q*‐statistic shows the presence of a highly significant level of heterogeneity, *I* ^2^ = 83.76 at *P*‐value <0.05. This indicates the need to use a random effect model for analysis. Therefore, based on random effect model analysis, the pooled prevalence/magnitude of women's knowledge of obstetric fistulas was 43.9% (95% CI 39.712, 48.002) (Figure 2). Subgroup analysis was performed to show the source of heterogeneity.

### 3.4 Sub‐group analysis for pooled prevalence of knowledge of obstetric fistula

The presence of heterogeneity was an indicator to carry out subgroup analysis to identify the source of heterogeneity. Study area, study design, study year, and sampling techniques were used as parameters. According to the findings based on setting, the magnitude of women's knowledge of obstetric fistulas was 40.8% in SNNPR, 41.6% in Amhara, 42.15% in Tigray, and 50% in Oromia and southwest region. According to community‐based cross‐sectional studies, the magnitude of women's knowledge of obstetric fistulas was 43.54% and 44.7% in institution‐based cross‐sectional studies, 42.15% in studies using a multi‐stage sampling technique, 43.04% in studies using a systematic random sampling technique and 50% in studies using a simple random technique. Again, the magnitude of women's knowledge of obstetric fistulas was 43% among studies conducted before or in 2020 and 45% among studies conducted after 2020. Therefore, the subgroup analysis result shows that study area, study years, sampling techniques, and study setting were not sources of heterogeneity (*P* < 0.001) (Table 2).

**TABLE 2:** Subgroup analysis for magnitude of knowledge of obstetric fistula.

Group	Number of studies	Prevalence (95% confidence interval)	*I* ^2^ (%)	*P*‐value
Region				
SNNP	1	40.8 (36.1, 45.49)	–	<0.01
Amhara	3	41.56 (34.25, 48.86)	88.42	<0.01
Oromia	1	50.00 (45.1, 54.9)	–	<0.01
Southwest	1	50.00 (45.1, 54.9)	–	<0.01
Tigray	1	42.15 (38.22, 46.09)	–	<0.01
Sampling technique				
Systematic random	5	43.04 (37.77, 48.31)	85.7	<0.01
Multi‐stage	1	42.15 (38.26, 46.09)	–	<0.01
Simple random	1	50.00 (45.10, 54.90)	–	<0.01
Year				
>2020	3	45.04 (36.43, 53.64)	91.42	<0.01
≤2020	4	43.05 (38.57, 47.53)	74.31	<0.01
Study design				
Community based	5	43.54 (38.53, 48.54)	85.27	<0.01
Institution based	2	44.73 (34.44, 55.02)	89.08	<0.01
Overall	7	43.86 (39.71, 48.00)	83.76	<0.01

### 3.5 Publication bias

**FIGURE 3:**
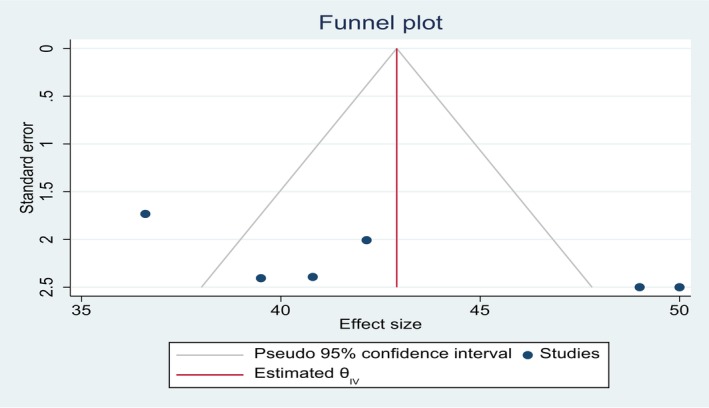
Funnel plot presentation of presence of publication bias.

There was publication bias, as shown by asymmetric funnel plots and a significant Egger test at *P*‐value = 0.0118 and Bigge's test at *P*‐value = 0.045 (Figure 3). To reduce and adjust publication bias, trim and fill analysis was performed.

### 3.6 Factors associated with knowledge of obstetric fistula

Factors associated with knowledge of obstetric fistulas by using variables reported from studies conducted in Ethiopia were “level of education,” “ever being pregnant,” “family planning history,” “previous place of delivery,” “heard obstetric complication,” “house distance from health institution,” “residence,” “having ANC,” “participate in conference,” and “having prior information about obstetric fistula.” Among these, “level of education,” “having ever pregnancy,” “FP history,” “having ANC in previous pregnancy,” “residence,” and “home far from heath institution” were significantly associated with knowledge of obstetric fistulas, while “participate in conference,” “having prior information about obstetric fistula,” “place of delivery,” and “heard about obstetric complication” were not significantly associated with knowledge of obstetric fistulas.

#### Level of education

**FIGURE 4:**
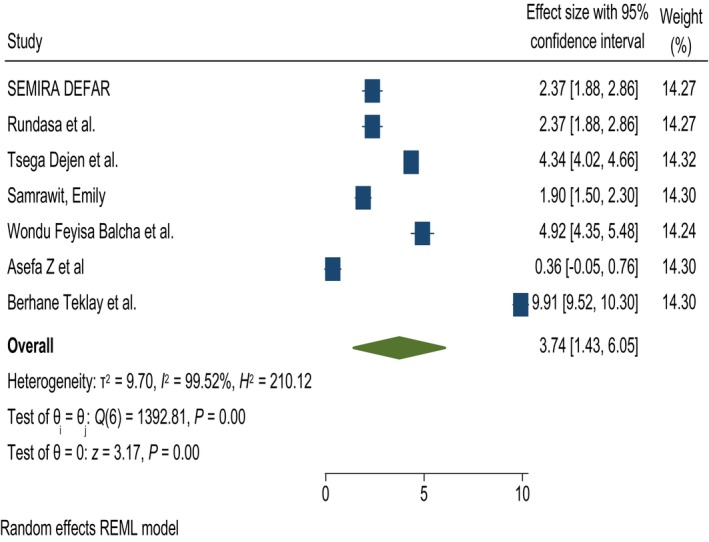
The association between level of education and knowledge of obstetric fistula. REML, random effect model.

Level of education was significantly associated with knowledge of obstetric fistula. Women who had a formal education were 3.74 times more likely to have good knowledge than those who have no formal education, with AOR 3.74, 95% CI = 1.43, 6.05, and test of heterogeneity *I* ^2^ = 99.52% (Figure 4).

#### Ever being pregnant

**FIGURE 5:**
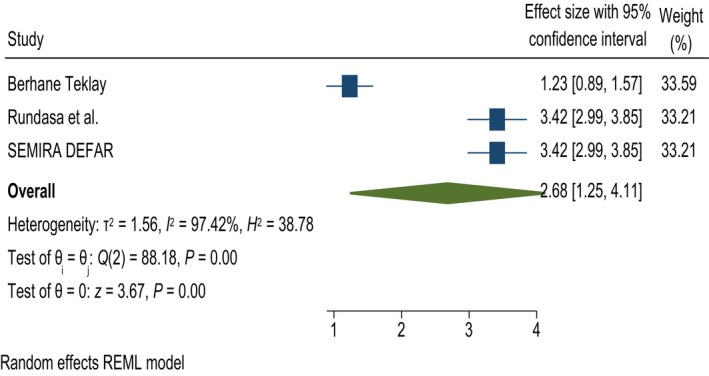
The association between being ever pregnant and knowledge of obstetric fistula. REML, random effect model.

In this study, having ever been pregnant was significantly associated with knowledge of obstetric fistulas. Women who had ever been pregnant were 2.68 times more likely to have good knowledge about obstetric fistulas than those who had never been pregnant; AOR = 2.68, 95% CI 1.25, 4.11, and *I* ^2^ = 97.42% (Figure 5).

#### Family planning history

**FIGURE 6:**
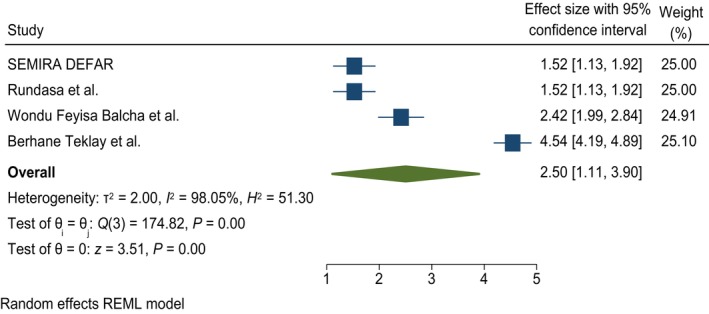
The association between family planning history and knowledge of obstetric fistula. REML, random effect model.

This systematic review and meta‐analysis shows that having a history of FP utilization was significantly associated with knowledge of obstetric fistulas. Women who had FP history were 2.5 times more likely to have good knowledge of obstetric fistulas; AOR = 2.5, 95% CI = 1.11, 3.9, and *I* ^2^ = 98.05% (Figure 6).

#### Home far from health institution

**FIGURE 7:**
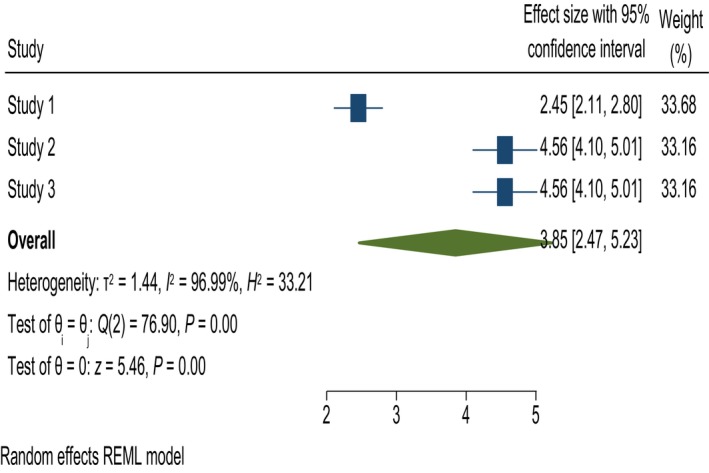
The association between home far from health institutions and knowledge of obstetric fistula. REML, random effect model.

In this study, home distance from the nearest health institution was significantly associated with knowledge of obstetric fistulas. A women whose house was ≤30 min from a health institution was 3.85 times more likely to have good knowledge than those whose house was more than 30 min from a health institution; AOR = 3.85, 95% CI = 2.47, 5.23, and *I* ^2^ 96.99% (Figure 7).

#### Residence

**FIGURE 8:**
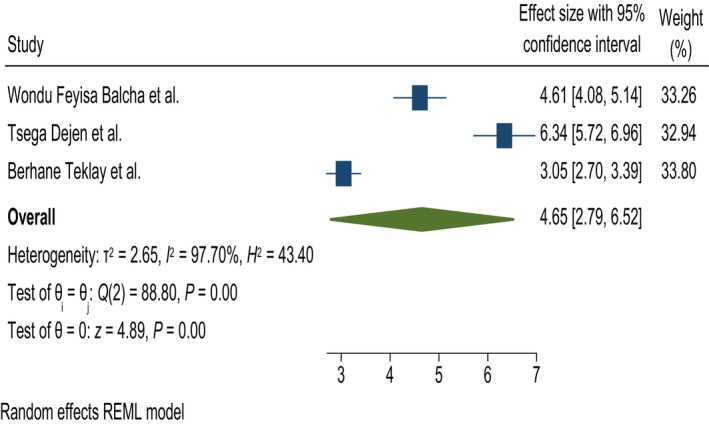
The association between residence and knowledge of obstetric fistula. REML, random effect model.

In this systematic review and meta‐analysis, residence was found to be significantly associated with knowledge of obstetric fistula. Women living in urban areas were 4.65 times more likely to have good knowledge than those who live in rural areas; AOR = 4.65, 95% CI = 2.79, 6.52, and *I* ^2^ = 97.7% (Figure 8).

#### Having previous antenatal care

**FIGURE 9:**
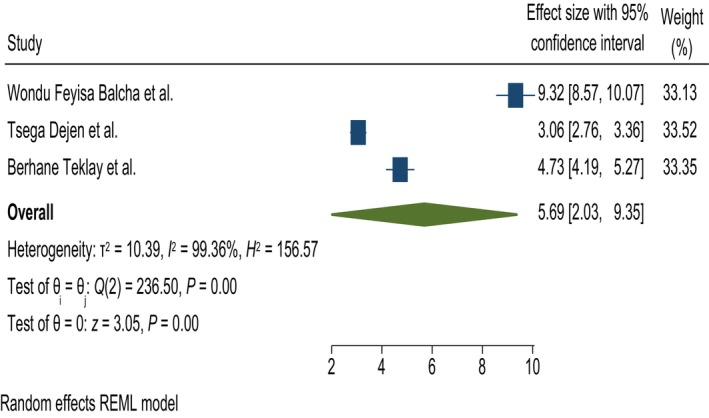
The association between having antenatal care (ANC) and knowledge of obstetric fistula. REML, random effect model.

In this study, having ANC in a previous pregnancy was significantly associated with knowledge of obstetric fistulas. Women who had previous ANC history were 5.69 times more likely to have good knowledge about obstetric fistulas than those who had no previous ANC; AOR = 5.69, 95% CI = 2.03, 9.3, and *I* ^2^ = 99.36% (Figure 9).

##### Place of delivery

In this study, place of delivery was not significantly associated with knowledge of obstetric fistula; AOR = 4.4, 95%, CI = −0.32, 9.13.

##### Participation in a conference

In this systematic review and meta‐analysis, participating in a conference was not significantly associated with knowledge of obstetric fistulas; AOR = 2.48, 95% CI = 0.28, 4.67.

##### Having prior information about fistulas

Having prior information about obstetric fistulas was not significantly associated with knowledge of obstetric fistulas; AOR = 11.73, 95% CI = −0.29, 23.76.

##### Having heard about obstetric complications

Again, hearing about obstetric fistulas was not significantly associated with knowledge of obstetric fistulas in this systematic review and meta‐analysis; AOR = 3.59, 95% CI = 0.28, 6.92.

## 4 DISCUSSION

The pooled prevalence of women's knowledge of obstetric fistulas and associated factors has been reported in different studies conducted on knowledge of obstetric fistulas in Ethiopia.^16^, ^21^, ^26^, ^27^ However, there is no nationwide published data on knowledge of obstetric fistulas in Ethiopia. Our study showed that 43.86% (39.7, 48) of women of reproductive age had good knowledge about obstetric fistulas in Ethiopia. This result is greater than that found in a study conducted in SSA (37.9%),^28^ Burkina (36.4%),^29^ Gambia (32.8%)^30^ and 12.8%.^31^ However, the prevalence in our study is lower than that found in studies conducted in Abakaliki, Ebonyi State, Nigeria (57.8%)^32^ and in a Nigerian Demographic Health Survey (52%).^33^ The difference might be due to socio‐demographic and socioeconomic variation across the country, variation in policy around engaging women and girls in education, and the quality of healthcare provision. The result of this study is in line with a study conducted in North Ghana (45.8%).^34^

“The level of education,” “ever being pregnant,” “family planning history,” “having ANC in a previous pregnancy,” “home far from a health institution,” and “residence” were significantly associated with knowledge of obstetric fistula. Women who had a formal education were 3.74 times more likely to have good knowledge than those who had no formal education (AOR = 3.74, 95% CI = 1.43, 6.05). This finding is consistent with a study conducted in SSA^28^ and a Demographic and Health Survey study conducted in Gambia^30^, ^31^ but inversely associated with a study conducted in Nigeria in which the awareness of fistulas was lower among those who had primary, secondary, and tertiary education compared with those with no formal education. Women who have ever been pregnant were 2.68 times more likely to have good knowledge about obstetric fistulas than those who had never been pregnant (AOR = 2.68, 95% CI 1.25, 4.11). This result is consistent with a study conducted in Nigeria^33^ and a study conducted in Gambia.^30^

Women who had FP history were 2.5 times more likely to have good knowledge of obstetric fistulas (AOR = 2.5, 95% CI = 1.11, 3.9). This finding differs from that of a study conducted in Nigeria.^33^ Women whose house was ≤30 min from a health institution were 3.85 times were more likely to have good knowledge than those whose house was more than 30 min from a health institution (AOR = 3.85, 95% CI = 2.47, 5.23). Women who had previous ANC history were 5.69 times more likely to have good knowledge about obstetric fistulas than those who had no previous ANC (AOR = 5.69, 95% CI = 2.03, 9.3), and women who live in urban areas were 4.65 times more likely to have good knowledge than those who live in rural areas (AOR = 4.65, 95% CI = 2.79, 6.52). This finding is in line with a study conducted in SSA^28^ but different from a study using the Nigerian Demographic Health Survey in which women living in rural areas were more likely to have good knowledge than those living in urban areas.^33^

## 5 CONCLUSION AND RECOMMENDATIONS

Less than half of women of reproductive age had good knowledge about obstetric fistulas. Attending formal education, urban residence, having ANC history, having FP history, home distance from health institution ≤30 min by foot and ever having been pregnant were positively associated with knowledge about obstetric fistulas. Therefore, it is better to provide health education and advocate for women (including young women) to be educated, to maximize access to health services, to enable women to utilize maternity and child health services, and to ensure women have contact with healthcare providers and live in close proximity to health institutions.

## AUTHOR CONTRIBUTIONS

All authors were involved in designing the first draft of this systematic review and meta‐analysis and efforts exerted to search online available articles, extract the data, analyze the extracted data, and write the reviewed manuscript. Again, all the authors were involved in quality assessment and in revising the subsequent draft of this manuscript. Finally, all authors read and approved the final draft of the manuscript. Aster Shiferaw, Getachew Tilaye Mihiret, and Mastewal Yechale Mihret were involved in revising the review comments and suggestions.

## CONFLICT OF INTEREST STATEMENT

The authors have no competing interests to declare.

## Supporting information


Appendix S1

## Data Availability

The data and materials used to generate this manuscript and the manuscript itself can be accessed from the corresponding author by email: astershiferaw21@gmail.com.
